# Versatile Direct (Hetero)Arylation Polymerization of Electro‐Deficient Unsubstituted Thiazolo[5,4‐d]Thiazole: A Tool to Lower the LUMO Level

**DOI:** 10.1002/marc.202500243

**Published:** 2025-05-13

**Authors:** Badr Jismy, Pablo Durand, Jasmine P. Jacob, Fanny Richard, Olivier Boyron, Benoit Heinrich, Bruno Schmaltz, Patrick Lévêque, Olivier Bardagot, Nicolas Leclerc

**Affiliations:** ^1^ Institute of Chemistry and Processes for Energy Environment and Health (ICPEES) CNRS University of Strasbourg 25 rue de Becquerel Strasbourg 67087 France; ^2^ Laboratoire de Physico‐Chimie des Matériaux et des Electrolytes pour l'Energie (PCM2E) Université de Tours EA6299 Tours 37200 France; ^3^ CNRS Institut de Science et d'Ingénierie Supramoléculaires Université de Strasbourg 8 alleé Gaspard Monge Strasbourg 67000 France; ^4^ CNRS Université de Strasbourg IPCMS UMR 7504 Strasbourg F‐67034 France; ^5^ CNRS Université de Lyon Laboratoire CP2M, UMR 5128 Villeurbanne 69100 France; ^6^ Laboratoire des Sciences de l'Ingénieur de l'Informatique et de l'Imagerie (ICube Research Institute) CNRS University of Strasbourg 23 rue du Loess Strasbourg 67037 France

**Keywords:** conjugated copolymers, direct (hetero)arylation polymerization, electron deficiency, thiazolo[5,4‐d]thiazole

## Abstract

A series of novel conjugated semiconducting polymers based on the unsubstituted thiazolo[5,4‐*d*]thiazole (TzTz) unit is synthesized using atom‐economic and environmentally friendly direct (hetero)arylation polymerization (DHAP). The versatility of the proposed polymerization conditions, employing a non‐chlorinated and moderately toxic solvent and cooperative palladium/copper bimetallic catalytic system, is demonstrated through the use of seven comonomers with varying electron‐withdrawing strength: 2,2′‐bithiophene (BT), 6,7‐difluoroquinoxaline (Qx), thieno[3,4‐*c*]pyrrole‐4,6(5H)‐dione (TPD), 5,6‐difluorobenzo[*c*][1,2,5]thiadiazole (BTD), isoindigo (IID), para‐azaquinodimethane (AQM) and 2,5‐dihydropyrrolo[3,4‐c]pyrrole‐1,4‐dione (DPP). The resulting TzTz‐based copolymers exhibit optical bandgaps between 1.5 and 2.0 eV with HOMO/LUMO energy levels spanning from −5.2/−3.3 eV to −5.4/−3.9 eV. They all show satisfactory thermal stability for electronic applications (Td = 300−360 °C). Notably, TzTz‐based copolymers are observed they generally exhibit improved backbone planarity and deeper LUMO levels than their thiophene derivatives. A synthesis tool to finely lower the LUMO levels of next‐generation A‐A’ copolymers in view of increasing the performance and air‐stability of doped organic electronics is believed to be provided by this work.

## Graphical abstract

An unsubstituted thiazolo[5,4‐*d*]thiazole (TzTz) unit is used to synthesize a series of novel semiconducting polymers via eco‐friendly direct arylation polymerization. These TzTz‐based copolymers, with various electron‐withdrawing comonomers, display optical bandgaps of 1.5–2.0 eV and strong thermal stability. The use of the unsubstituted TzTz unit improves backbone planarity and lowers LUMO levels, which are interesting features for organic electronics.

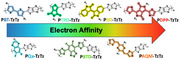

## 1 Introduction

Organic electronic devices such as logic circuits and thermoelectric generators require both p‐ and n‐type materials to operate efficiently. However, the current literature is dominated by the development of p‐type polymers, while the development of high‐performance and air‐stable electron‐transporting n‐type polymers is still lagging behind, especially in terms of operational stability and mobility/conductivity.^[^ ^1^ ^]^

Many n‐type polymers rely on the chemical architecture based on the alternation of electron‐rich and electron‐withdrawing units (donor/acceptor (D‐A) copolymers).^[^ ^2^ ^]^ The archetypal D‐A n‐type polymer is PNDI‐T_2_ (also known as N2200) with the naphthalene‐diimide unit as the electron‐deficient unit.^[^ ^3^ ^]^ The D‐A approach is highly popular as it enables easy synthetic routes and fine control over the energy levels of both the highest occupied molecular orbital (HOMO) and the lowest unoccupied molecular orbital (LUMO). However, the copolymerization with an electron‐rich unit, the 2,2′‐bithiophene (BT) in the PNDI‐T_2_ case, severely limits the ability to deepen the LUMO level, yet crucial to achieving stable devices operating in ambient conditions.^[^ ^4^, ^5^ ^]^ In addition, the large variation of electronic density between the D and A units localizes the HOMO and LUMO on each unit, respectively,^[^ ^6^ ^]^ and limits (bi)polaron delocalization when doped.^[^ ^6^, ^7^ ^]^ The limited library of full‐acceptor A‐A’ n‐type polymers available to date suggests that there is still work to do to develop innovative electron‐deficient units and the associated chemistry.^[^ ^8^ ^]^ In particular, many highly electron‐deficient units have proved difficult to couple via traditional cross‐coupling reaction conditions with transition metals.^[^ ^9^ ^]^ Moreover, due to highly polar and planar units, electron‐deficient units challenge the balance between solubility and processing (most of the electron‐deficient units are heteroaromatic units leading to weak bond networks and decreasing the solubility).^[^ ^10^, ^11^ ^]^ In summary, the emergence of conjugated polymer applications calls for the maturation of comonomers with the following criteria: low cost, ease of functionalization, preservation of backbone planarity, and increased electron deficiency, all of which are essential for the synthesis of scalable high‐performance A‐A’ copolymers.

**Scheme 1:**
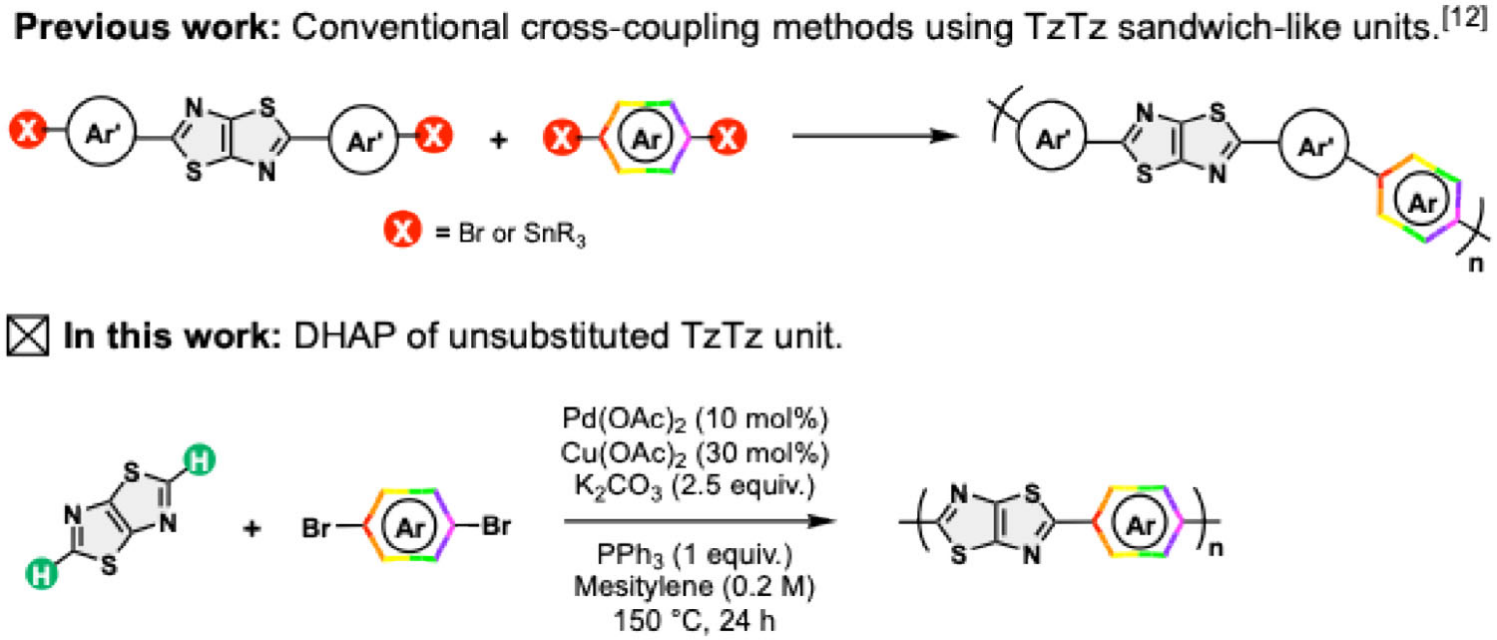
Overview of different strategies for preparing TzTz‐based conjugated polymers and structure of investigated copolymers.

Thiazolo[5,4‐*d*]thiazole (TzTz) was reported in the early 60′s by Ketcham and coworkers (**Scheme** **1**).^[^ ^12^ ^]^ Since TzTz has been investigated in several fields of application such as medicinal chemistry,^[^ ^13^ ^]^ chemoselective molecular sieving,^[^ ^14^ ^]^ fluorescent optical sensors,^[^ ^15^ ^]^ and all‐organic optoelectronic devices.^[^ ^16^, ^17^, ^18^, ^19^, ^20^ ^]^ TzTz is considered a weak electron‐deficient unit.^[^ ^18^ ^]^ It consists of two fused five‐membered rings and when coupled with heteroaromatic units, it leads to a highly planar conjugated structure, owing to low steric hindrance and potential weak heteroatom interactions. These interactions, arising from the non‐bonding electron pair on nitrogen atoms,^[^ ^21^ ^]^ contribute to conformation locking.^[^ ^22^, ^23^ ^]^ Copolymers with planar backbones tend to exhibit high transfer integral overlaps and strong π‐π stacking, favoring hopping transport in the intra‐ and inter‐chain directions, respectively, which results in high charge carrier mobilities.^[^ ^24^, ^25^, ^26^ ^]^ Besides, thanks to its reduced number of CH groups, TzTz is compatible with sustainable direct (hetero)arylation polymerization (DHAP) methods. Thus, the TzTz unit emerges as a promising, more electron‐withdrawing alternative to (bi)thiophenes for the synthesis of ambipolar and n‐type copolymers.

Importantly, traditional synthetic routes to TzTz derivatives predominantly yield compounds in which the TzTz core is sandwiched between two aromatic units (Scheme 1). These conventional strategies typically involve cyclization reactions, leading to polyaromatic systems where the choice of neighboring units of the TzTz moiety is limited. The presence of these additional aromatic rings often restricts the versatility of the TzTz building block, reducing its potential for fine‐tuning (opto)electronic properties.^[^ ^18^ ^]^

**Figure 1:**
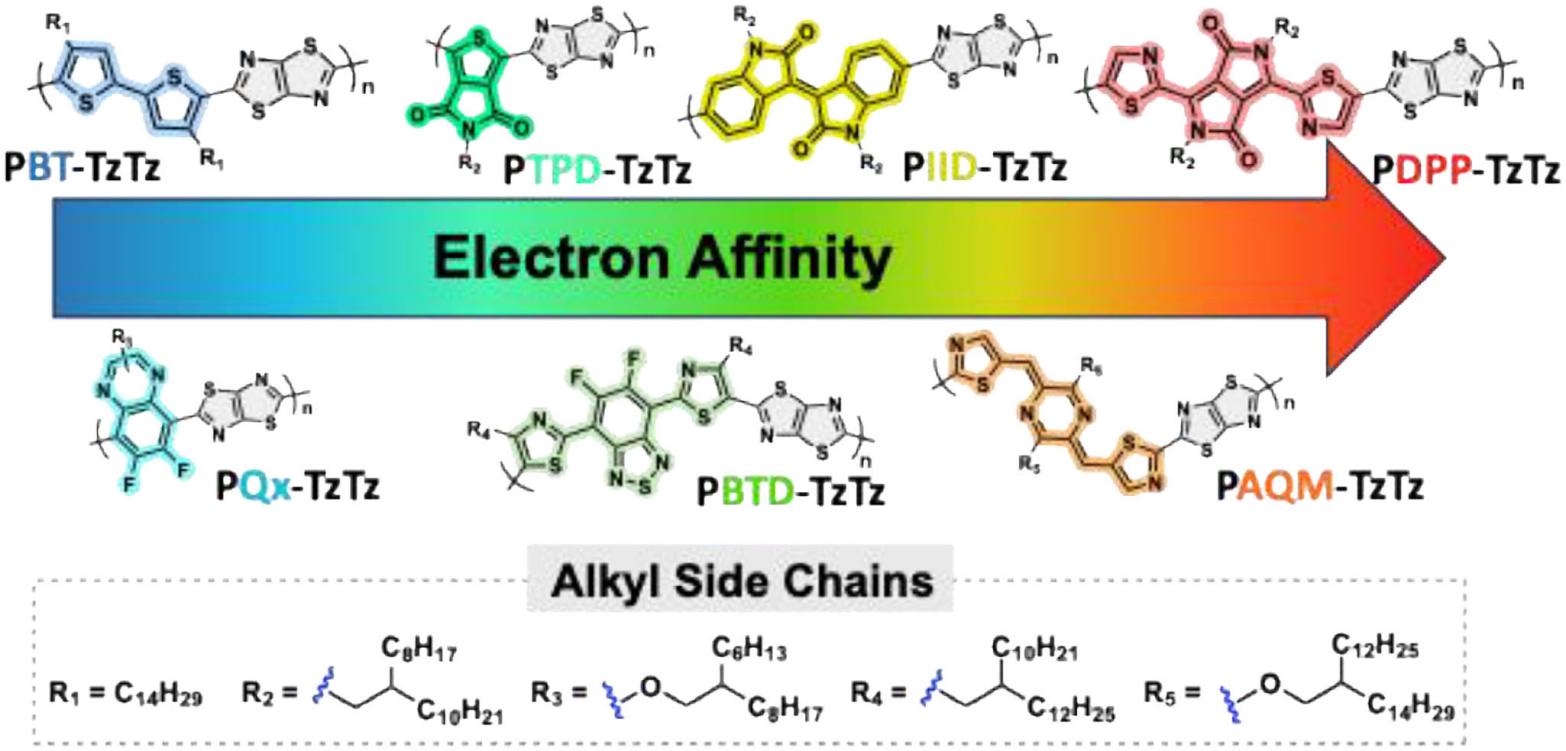
Chemical structures of the TzTz‐based copolymers. The arrow represents the increasing evolution of the electronic affinity of these copolymers.

In this article, we present the development of unsubstituted TzTz as a key building block for the synthesis of novel A/A’ copolymers. The innovations of this work lie in i) the use of the unsubstituted TzTz unit as a standalone comonomer, meaning that the TzTz core is *not* sandwiched between any spacing units, similarly to its thieno[3,2‐*b*]thiophene (TT) parent,^[^ ^27^, ^28^ ^]^ and ii) the report of the synthetic conditions to copolymerize it with a wide range of electron‐withdrawing comonomers via DHAP (Scheme 1). The use of DHAP decreases the number of synthetic steps while eliminating the use of toxic reagents/byproducts, providing a low‐cost alternative to conventional cross‐coupling polycondensations such as Stille and Suzuki couplings.^[^ ^29^, ^30^ ^]^ In this work, we show the versatility of our approach by combining the unsubstituted TzTz unit with seven comonomers of variable electron‐withdrawing strength: namely 2,2′‐bithiophene (BT), 6,7‐difluoroquinoxaline (Qx), thieno[3,4‐c]pyrrole‐4,6(5H)‐dione (TPD), 5,6‐difluorobenzo[c][1,2,5]thiadiazole flanked with two alkylated thiazole units (BTD), isoindigo (IID), para‐azaquinodimethane (AQM) and 2,5‐dihydropyrrolo[3,4‐c]pyrrole‐1,4‐dione (DPP), the last two including two unsubstituted thiazole spacers (**Figure** **1**). Variations of the electron‐withdrawing coupling partner allow us to assess the efficiency of the proposed polymerization conditions. They also allow us to explore, for the first time, the structure‐property relationships of TzTz‐based copolymers. Note that care has been taken to use mesitylene, a moderately toxic non‐chlorinated solvent, and a cooperative Pd/Cu dual‐catalyst system^[^ ^31^ ^]^ to foster the transfer of the reported method to large‐scale synthesis in industrial environments. We believe this work makes a significant contribution to the advancement of TzTz‐based polymers for the future design of high‐performance electron‐withdrawing conjugated polymers finding applications, particularly in doped organic electronics.

## 2 Results and Discussion

**Figure 2:**
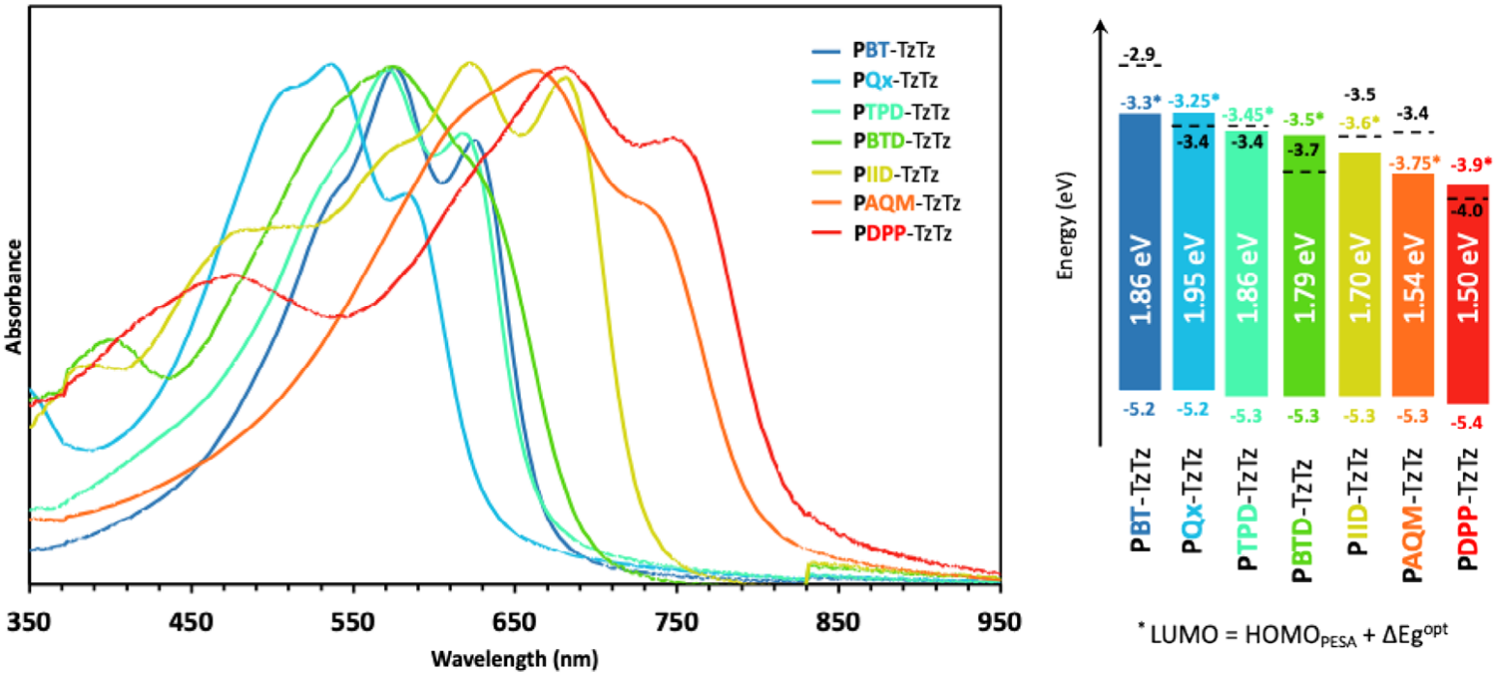
Left. UV–vis absorbance spectra of all copolymers in spin‐coated thin films. Right. Schematic energy diagram of all copolymers. Optical bandgaps (∆Eg^opt^) are reported in white. HOMO levels are extracted from photoelectron spectroscopy in air (PESA) measurements and used to calculate LUMO* levels (noted with stars) following the equation LUMO* = HOMO_PESA_ + ∆Eg^opt^. LUMO_CV_ levels reported in black with dashed lines are extracted from the CV measurements.

The thiazolo[5,4‐*d*]thiazole (TzTz) core is synthesized following the standard Ketchman route using furan‐2‐carbaldehyde as a precursor (see details in Supporting Information).^[^ ^32^ ^]^ In this study, we present how to use unsubstituted TzTz as a building block, without two sandwiching aromatic units. The unsubstituted TzTz monomer is afforded by oxidizing the 2,5‐difuryl‐TzTz into the dicarboxylic acid followed by a further decarboxylation.^[^ ^32^ ^]^ TzTz has a planar geometry which forms a white crystalline powder.^[^ ^33^ ^]^ We copolymerize this unsubstituted TzTz by DHAP with seven comonomers of increasing electron‐withdrawing strength, resulting in seven new copolymers: PBT‐TzTz, PQx‐TzTz, PTPD‐TzTz, PBTD‐TzTz, PIID‐TzTz, PAQM‐TzTz and PDPP‐TzTz (**Figure** **2**). These seven comonomers are functionalized with two bromine atoms and synthesized following procedures previously reported in the literature (see details in Schemes S1 and S2, Figures S1–S16, Supporting Information). The 5,5′‐dibromo‐4,4′‐didodecyl‐2,2′‐bithiophene (BT‐C_14_H_29_) is used as a reference electron‐donating unit in order to evaluate our polymerization conditions against a previously published Stille cross‐coupling approach.^[^ ^25^ ^]^ Concerning the side chains, we used alkyl side chains that are commonly described for each of these comonomers without seeking to innovate at this stage, in order to allow direct comparisons with equivalent thiophene‐based materials (see Table 2).

For the development of TzTz‐based copolymers, the DHAP conditions are selected based on the chemical considerations associated with the CH‐activation of the single thiazole unit. Indeed, the C2‐position of thiazole is relatively electron‐deficient and acidic (pKa ≈29.5) due to the proximity of the two heteroatoms.^[^ ^34^, ^35^ ^]^ Furthermore, the acidity of the C2‐position can be increased by copper complexation of the thiazole nitrogen, thus facilitating the deprotonation step. For this purpose, the C2 arylation of the thiazole unit is selectively preferred when an appropriate base and Pd/Cu(I) co‐catalytic systems are applied.^[^ ^36^ ^]^ Inspired by this literature analysis, we adapted the DHAP conditions to synthesize the seven new TzTz‐based copolymers (Scheme 1).

After polymerization, all polymers are purified by Soxhlet extraction and treated with a Pd scavenger. Each fraction is characterized by high‐temperature size‐exclusion chromatography (SEC) in 150 °C trichlorobenzene (TCB, see **Table** **1** and Figures S17–S28, Supporting Information). Table S1 (Supporting Information) reports the molar masses and distribution of the mass amount of polymer extracted in each fraction. The relatively low molar masses achieved (M_n_ < 14.5 kg mol^−1^) are rationalized by the lower solubility of thiazole‐based polymers compared to their thiophene‐based analogs. Indeed, the replacement of the C−H bond by a nitrogen atom decreases the local steric hindrance. Combined with possible noncovalent interactions between the nitrogen atom of the thiazole and the heteroatoms of adjacent units (sulfur in thiophene‐based rings, oxygen in keto‐based units, …), it is a strong enhancement of the polymer backbone planarity leading to rapid aggregation and precipitation in solution.^[^ ^37^, ^38^, ^39^ ^]^ As a comparison, McCullough and coworkers polymerized by Stille cross‐coupling the PBT‐TzTz polymer with linear dodecyl side chains and could not characterize it by SEC due to insolubility.^[^ ^25^ ^]^ They had to add two extra alkylated thiophenes to get a soluble polymer, reaching an M_n_ of 7.2 kg mol^−1^, two‐fold lower than the M_n_ estimated for the PBT‐TzTz presented here. This comparison suggests that our polymerization conditions are well suited to cross‐couple the unsubstituted TzTz unit to BT. In addition, the acceptable molar masses of PBTD‐TzTz (M_n_ = 10.3 kg mol^−1^), involving only C‐C couplings between thiazole units, further supports that these polymerization conditions are well suited for the synthesis of A/A’ electron‐deficient copolymers. Note that we believe that the SEC data, even at high temperatures, are not appropriate to characterize most of the presented copolymers, as the higher masses are probably filtered off before injection in the SEC column. The similar measured molar masses between fractions supposed to be of different lengths (following Soxhlet fractionation, see PQx‐TzTz, PIID‐TzTz, and PAQM‐TzTz), as well as the high dispersity, are indirect evidence of the nonoptimal conditions for molar mass characterization and hence of a probable underestimation of the highest molar masses by SEC.

**Table 1:** Molar masses and dispersity estimated from SEC in 150 °C TCB for the chlorobenzene fraction and thin films' optical properties.

Polymer	SEC Properties		Thin‐Film UV–vis Absorbance		
	M_n_/M_w_ [kg/mol]	Đ	λ_max_ [nm]	λ_onset_ [nm]	∆Eg^opt^ [eV]
**PBT‐TzTz**	14.5/37.6	2.6	556	665	1.86
**PQx‐TzTz**	3.3/7.1	2.1	536	635	1.95
**PTPD‐TzTz**	7.3/13.9	1.9	571	666	1.86
**PBTD‐TzTz**	10.3/16.4	1.6	577	691	1.79
**PIID‐TzTz**	1.5/6.6	4.4	624, 684	730	1.70
**PAQM‐TzTz**	3.9/7.4	1.9	664	804	1.54
**PDPP‐TzTz**	3.9/8.3	2.1	680	825	1.50

In order to compare polymers with each other, we focus on the characterization of chlorobenzene fractions only.

The copolymers are birefringent solids whose thermal properties are investigated through thermogravimetric analysis (TGA) and differential scanning calorimetry (DSC) measurements. All copolymers are thermally stable at least up to 300 °C under nitrogen, making them suitable for applicative uses (see decomposition temperatures in Table S2, Supporting Information). Amongst all polymers, only PIID‐TzTz (phase change below 40 °C) and PBT‐TzTz show thermal transitions (see Figures S25 and S26, Supporting Information). PBT‐TzTz exhibits a semi‐crystalline phase at room temperature (RT), which undergoes a solid phase transition just above RT. It then melts into a fluid, birefringent liquid‐crystalline phase between 90 and 110 °C. This behavior is comparable to that of its all‐thiophene analog, PBTTT, with the key difference that PBT‐TzTz remains in the liquid‐crystalline phase until it begins to degrade at ≈320 °C. Additional small‐ and wide‐angle X‐ray scattering (SWAXS) measurements on PBT‐TzTz powder reveal lamellar and π‐stacking distances that align well with those of PBTTT‐C_14_ (23.1 Å < *d*_lam_ < 24.1 Å, *h*_π_ = 3.61 Å), suggesting that the solid‐state structures of the two polymers are similar, likely featuring interdigitated side chains in the semi‐crystalline state (see Figure S31, Supporting Information).^[^ ^40^ ^]^ SWAXS diffraction studies of the other copolymers are beyond the scope of this article.

The UV–vis absorbance properties of all copolymers are studied in chlorobenzene solutions as a function of the temperature (from RT to 100 °C) as well as in thin films (see Table 1, Figure 2; Figures S32–S39, Supporting Information). All seven copolymers exhibit a broad (150 to 200 nm wide) absorbance band in the visible range. Notably, in solution at RT, the absorbance spectra of all copolymers resemble the ones of thin films, suggesting strong aggregation (see Figures S32–S39, Supporting Information). The aggregation of the polymers in RT solutions is supported by the presence of distinct vibronic transitions and characteristics of high molecular order.^[^ ^41^ ^]^ These observations further support the highly planar nature of TzTz‐based copolymers, limiting their solubility. For comparison, Figure S39 (Supporting Information) shows the absorbance spectrum of PBTTT‐C_14_, a thiophene analog of PBT‐TzTz. PBTTT‐C_14_ is well soluble at 25 °C in chlorobenzene (no aggregates), as indicated by its absorbance profile having a Gaussian shape and exhibiting a significant blue‐shift compared to its solid‐state absorbance profile in thin films. This comparison underlines the gain in backbone planarity and propension of the polymer to self‐assemble induced by the substitution of thiophene for thiazole units.

Looking at the onset of absorbance, no direct relationship between the optical bandgap and the electron‐withdrawing strength of the comonomers is observed across the TzTz‐based polymer series (Figure 2). This is justified by the several other factors influencing the bandgap of conjugated polymers, including the extent of hybridization of the frontier orbitals (which depends on the energy levels of the individual building blocks of the backbone), as well as intramolecular conformations and intermolecular stacking.^[^ ^42^ ^]^

Nevertheless, two distinct families of polymers can be identified. The first is a high bandgap family, with an optical bandgap (∆Eg^opt^) greater than 1.85 eV, achieved when using a weak electron‐donating comonomer (BT) and relatively weak electron‐withdrawing comonomers (Qx and TPD). The highest optical bandgap of the series is measured for PQx‐TzTz, with a value of 1.9 eV, comparable to poly(3‐hexylthiophene) P3HT.^[^ ^43^ ^]^ The second is a moderate‐ and low‐bandgap family, with ∆Eg^opt^ ranging from 1.5 to 1.8 eV. Narrower bandgaps were expected due to the incorporation of stronger electron‐accepting units as comonomers (BTD, IID, AQM, and DPP). The lowest optical bandgap of the series is obtained for PDPP‐TzTz, with a value of 1.5 eV. This latter shows an absorbance onset in the near‐infrared (NIR) region ≈850 nm.

The polymer energy levels in thin films are investigated using cyclic voltammetry (CV, Figures S36–S42, Supporting Information) versus ferrocene/ferrocenium (Fc/Fc^+^), as well as PESA (Figure 1; Figure S47, Supporting Information). The HOMO levels measured by PESA are all within a short range from −5.2 to −5.4 eV following a clear trend: the larger the electron‐withdrawing strength of the comonomer, the deeper the HOMO level is. [Chem. Phys. Lett. 1994, 217, 507–512] Nonetheless, as expected, the greatest variation within the series is found in the LUMO levels. The values of the LUMO levels, calculated by adding the optical bandgap to the HOMO measured by PESA, scale from −3.3 to −3.9 eV. The LUMO values extracted from CV measurements range from −2.9 to −4.0 eV. Again, a trend can be observed, with deeper LUMOs as the TzTz partner becomes more electro‐deficient. While the three high‐bandgap copolymers may be generally considered electron‐donating materials, the four copolymers with moderate to low bandgaps seem to exhibit deeper LUMO levels (below −3.5 eV), which could suggest their classification as electron‐withdrawing materials.

Comparisons with equivalent structures composed of thiophene units instead of thiazole units enable us to rationalize the impact of TzTz on the optoelectronic properties (**Table** **2**). PBT‐TzTz can be directly compared with PBTTT,^[^ ^28^ ^]^ PBTD‐TzTz with PBTD‐TT (commonly named PF_2_, TT instead of TzTz and BTD flanked by thiophenes instead of thiazoles),^[^ ^44^ ^]^ PIID‐TzTz with the PIID‐TT,^[^ ^45^ ^]^ PAQM‐TzTz with PAQM2T‐TT (TT instead of TzTz and AQM flanked by thiophenes instead of thiazoles),^[^ ^46^ ^]^ and finally PDPP‐TzTz with PDPP‐TT (TT instead of TzTz and DPP flanked by thiophenes instead of thiazoles).^[^ ^44^ ^]^ Several clear and consistent trends emerge from these comparisons. In all cases, the optical bandgap slightly increases when thiazole/thiazolothiazole units replace thiophenes (One can notice the slightly greater impact on the DPP‐based derivatives). This result is somewhat counter‐intuitive, given the generally more planar conformation of TzTz‐based copolymers, as calculated from the DFT at the B3LYP function with the 6–311G basis, which could let us think of a larger conjugation length typically associated with narrower bandgaps (Table 2; Figures S48–S55, Supporting Information). However, this trend is in full agreement with previous reports and is likely attributable to a reduced extent of the conjugation length in the imine‐based aromatic units.^[^ ^47^, ^48^ ^]^ Another notable trend is the concomitant lowering of the energy levels of both HOMO and LUMO, with the exception of the AQM‐based copolymer. This effect, which has been commonly observed in various thiazole‐based materials, is attributed to the electron‐deficient nature of the thiazole unit, due to the presence of the imine group.^[^ ^49^, ^50^, ^51^ ^]^ This comparison hence allows us to confidently state that the TzTz unit is a promising building block to foster the design of novel ambipolar and n‐type conjugated copolymers.

**Table 2:** Comparison of optoelectronic properties of thiophene‐ and thiazole‐based copolymers.

Polymer		λ_onset_ [nm]	∆Eg^opt^ [eV]	HOMO_PESA_ [eV]	LUMO [eV]^a^ ^)^	Calculated θ Angle [°]^b^ ^)^
**PBT‐**	**TzTz**	665	1.86	−5.2	−3.3	9
	**TT^[^** ^28^ **^]^**	652	1.90	−4.8	−2.9	65
**PQx‐**	**TzTz**	635	1.95	−5.2	−3.25	0
	**TT**	–	–	–	–	0
**PTPD‐**	**TzTz**	666	1.86	−5.3	−3.45	0
	**TT**	–	–	–	–	1
**PBTD‐**	**TzTz**	691	1.79	−5.3	−3.5	15
	**TT** ^[^ ^44^ ^]^	756	1.64	−5.1	−3.45	29
**PIID‐**	**TzTz**	730	1.70	−5.3	−3.6	14
	**TT** ^[^ ^45^ ^]^	800	1.55	–	–	18
**PAQM‐**	**TzTz**	804	1.54	−5.3	−3.75	0
	**TT** ^[^ ^46^ ^]^	848	1.46	−5.6	−4.1	0
**PDPP‐**	**TzTz**	825	1.50	−5.4	−3.9	40
	**TT** ^[^ ^44^ ^]^	1019	1.21	−5.1	−3.9	9

^a)^ LUMO levels are calculated using the following equation LUMO = HOMO_PESA_ + ∆Eg^opt^;

^b)^ Torsion angle calculated between the two planes formed by the units at the ends of the molecule (geometry optimized by DFT at B3LYP/6‐311G level), indicative of the relative deviation from a perfect planarity. The larger the angle is, the less planar the repeat unit is (Figures S48–S55, Supporting Information).

## 3 Conclusion

In this study, we introduce the “naked” unsubstituted thiazolo[5,4‐d]thiazole (TzTz) unit as a versatile building block for the development of high‐performance ambipolar and n‐type conjugated copolymers. By employing a DHAP route, which reduces synthetic steps and eliminates toxic reagents, we successfully synthesize a range of copolymers with varied electron‐deficient comonomers. The copolymers exhibit optical bandgaps ranging from 1.5 to 1.9 eV. Notably, we observe that TzTz‐based copolymers generally exhibit improved backbone planarity and slightly deeper LUMO levels than their thiophene derivatives. Overall, TzTz is shown to be a versatile and effective building block for the design of a broad range of organic semiconductors with promising applications in organic optoelectronic devices. Further optimization of polymerization conditions and comonomer selection, including an investigation of the side chain nature, could result in enhanced performance in the device and scalability of these materials.

## Conflict of Interest

The authors declare no conflict of interest.

## Supporting information



Supporting Information

## Data Availability

The data that support the findings of this study are available on request from the corresponding author.
