# Supplementary material for: Versatile Direct (Hetero)Arylation Polymerization of Electro‐Deficient Unsubstituted Thiazolo[5,4‐d]Thiazole: A Tool to Lower the LUMO Level
Source: Macromol Rapid Commun. 2025 May 13;46(16):2500243. doi: 10.1002/marc.202500243 (PMC12360140; doi:10.1002/marc.202500243)
Supplement: Supplementary file 1 — Supporting Information [file MARC-46-2500243-s001.docx]

Supporting Information

### Versatile direct (hetero)arylation polymerization of electro-deficient unsubstituted thiazolo[5,4-d]thiazole: a tool to lower the LUMO level

Badr Jismy, Pablo Durand, Jasmine P. Jacob, Fanny Richard, Olivier Boyron, Benoit Heinrich, Bruno Schmaltz, Patrick Lévêque, Olivier Bardagot and Nicolas Leclerc.

**Contents**

1. **General procedures and instruments**
2. **Materials and synthesis**
3. **SEC data**
4. **Thermal characterizations**
5. **UV-Vis Absorbance**
6. **Cyclic Voltametry**
7. **PESA**
8. **DFT calculations**
9. **References**
10. **General procedures and instruments**

**Materials.** Pd(OAc)_2_, Cu(OAc)_2_, PPh_3_, K_2_CO_3_, mesitylene and other chemicals were received from commercial suppliers and used without further puriﬁcation. 5,8-Dibromo-6,7-difluoro-2-((2-hexyldecyl)oxy)quinoxaline was purchased from Ossila and was then purified by chromatography (eluent: petroleum ether) giving off-white solid. 5,5’-Dibromo-4,4’-bis(tetradecyl)-2,2’-bithiophene,^[1]^ 1,3-dibromo-5-(2-octyldodecyl)-4*H*-thieno[3,4-*c*]pyrrole-4,6(5*H*)-dione^[2]^ and 2,5-bis-(2-octyl-dodecyl)-3,6-bis-thiazol-2-yl-2,5-dihydro-pyrrolo[3,4-c]pyrrole-1,4-dione^[3]^ were prepared according to the literature methods.

**NMR analysis.** ^1^H, ^13^C NMR spectra were recorded on a Bruker 400 UltrashieldTM 400 MHz NMR spectrometer, with an internal lock on the ^2^H-signal of the solvent (CDCl_3_).

**UV-visible measurements.** Absorbance spectra in solution and in thin films were recorded on a Shimadzu UV-2600 spectrophotometer. In solid state, the absorption spectra were measured on thin films drop-casted on glass substrates from a 0.5 mg/mL *o*-dichlorobenzene solution of organic polymers. In-situ UV-vis temperature dependent experiments in solid-state were performed using an Agilent Cary Spectrophotometer.

**Size exclusion chromatography.** Size Exclusion Chromatography (SEC) measurements were performed with Viscotek system, from Malvern Instruments, that incorporates a differential refractive index, a dual light-scattering detector and a viscosimeter. 1,2,4-Trichlorobenzene was used as the mobile phase at a flow rate of 1 mL/min at 150°C. It was stabilized with 2,6- di(tert-butyl)-4-methylphenol (200 mg L-1). The polymer was injected at a concentration of 1 mg/mL. The separation was carried out on three Agilent columns (PLgel Olexis from Agilent Technologies, 300 mm × 7.5 mm, 13µm) protected by a guard column (PL gel 5 μm). Columns and detectors were maintained at 150°C. The OmniSEC software version 5.2 was used for data acquisition and analysis. The molar mass distributions were calculated with a calibration curve based on narrow polystyrene standards (Polymer Standard Service, Mainz), using only the refractometer detector.

**Thermal properties.** TGA measurements were performed with TA Instruments Q50 from instrument. DSC measurements were performed with TA Instruments Q1000 instrument, operated at scan rate of 5o C/min on heating and on cooling.

**Cyclic voltammetry.** Oxidation and reduction potentials were determined by cyclic voltammetry with a conventional 3-electrode system using a BioLogic potentiostat equipped

with a platinum micro disk (2 mm2) working electrode and a platinum wire counter electrode. Potentials were calibrated versus the saturated calomel electrode (SCE) at a conventional scan rate of 100 mV/s. Recrystallized tetrabutylammonium hexafluorophosphate (Bu_4_NPF_6_) was used as the supporting electrolyte (0.1 M) in distilled and anhydrous acetonitrile. Acetonitrile was distilled from CaH_2_ under a nitrogen atmosphere. The ferrocene/ferrocenium couple was used as an internal reference. HOMO and LUMO levels were calculated following these equations: HOMO = -(4.4 + E_onset_ ox. vs SCE) eV and LUMO = -(4.4 + E_onset_ red. vs SCE) eV.

**PESA.** Photo-Electron Spectroscopy in Air (PESA) has been performed on a AC-2 Model from Riken Instruments. UV photons are emitted from a deuterium lamp, then monochromated by a grating spectrometer and finally focused on the sample film. The photoelectrons emitted by the sample are detected by an open counter. When the sample's surface is bombarded with a slowly increasing amount of UV energy, photoelectrons start to emit at a certain energy level which corresponds to the photoelectron work function.

**Structural and thermal analysis.** The Small- and Wide-angle X-ray scattering (SWAXS) patterns were obtained with transmission Guinier-like geometry. A linear focalized monochromatic Cu Kα1 beam (λ = 1.54056 Å) was obtained by using a sealed-tube generator (600 W) equipped with a bent quartz monochromator. The sample was filled in a sealed cell with an adjustable path. The sample temperature was controlled within ±0.1 °C, and exposure times were equal to 24 h. The patterns were recorded on image plates scanned by an Amersham Typhoon IP with 25 μm resolution (periodicities up to 120 Å). The I(q) profiles were obtained from images by using home-developed software. Optical textures were observed with a Leitz Orthoplan polarizing microscope equipped with a Mettler FP82 hot stage and a FP80 unit. The DSC measurements were carried out with a Q1000 apparatus of TA Instruments, operated at a scanning rate of 5 °C/min, on heating and on cooling. The TGA measurements were carried out with a Q50 apparatus of TA Instruments, at a scanning rate of 5 °C/min and with nitrogen as purge gas.

**DFT.** All Density Functionnal Theory calculations were performed using Spartan 10 at the B3LYP/6-311+G* level of theory in vacuum. To keep the computational time within a reasonable range, the alkyl chains were replaced by ethyl groups and the alkoxy chains by methoxy groups. The planarity was estimated by the measurement of the angle between the two green planes at the end of the molecules allowing a direct comparison between the TzTz molecules and their TT analogues.

1. **Materials and synthesis**

**Synthesis of Benzothiadiazole-based monomer**

**Scheme S1.** Synthetic route to Benzothiadiazole-based monomer

**1-Bromo-4-octyltetradecan-2-one (4**): was prepared according to the literature methods.^[4,5]^

**4-Octyl-1-thiocyanatotetradecan-2-one (5):** In a 100 mL RBF was added 1-bromo-4-

octyltetradecan-2-one (4) (2 g, 5 mmol), KSCN (0.72 g, 7.5 mmol) and 50 mL of ACN. The mixture was stirred at room temperature for 24h then the solvent was eliminated. The residue was taken in PE and filtered. The filtrate was evaporated giving 1.85 g of colorless oil. Yield = 95 %. ^1^H NMR (CDCl3, 400 MHz) *δ*: 4.00 (s, 2H), 2.42 (d, 2H, *J* = 6.6 Hz), 1.84 (m, 1H), 1.17 (m, 32H), 0.79 (t, 6H); ^13^C NMR (CDCl3, 100 MHz) δ: 201.2, 111.3, 46.0, 44.2, 33.9, 33.7, 31.7, 31.7, 29.7, 29.7, 29.5, 29.5, 29.4, 29.4, 29.2, 29.1, 26.4, 26.4, 22.5, 22.5, 13.9, 13.9.

**2-Bromo-4-(2-octyldodecyl)thiazole (6):** In a 50 mL RBF was added 4-octyl-1-thiocyanatotetradecan-2-one (5) (1 g, 2.6 mmol) and 10 mL of 33 % HBr in AcOH. The solution was stirred at RT for 24h then poured into ice, neutralized with concentrated NaOH keeping the temperature below 10 °C and extracted with DCM. The combined organic layer were washed with brine and purified with silica gel chromatography (PE:DCM 10%) giving 0.7 g of yellowish oil. Yield= 60 %.

^1^H NMR (CDCl_3_, 400 MHz) *δ*: 6.76 (s, 1H), 2.63 (d, 2H, *J* = 6.97 Hz), 1.79 (m, 1H), 1.23 (m, 32H), 0.86 (t, 6H) ^13^C NMR (CDCl_3_, 100 MHz) δ: 157.6, 134.5, 117.3, 44.2, 37.6, 36.4, 33.3, 32.0, 32.03, 30.1, 30.1, 29.8, 29.8, 29.7, 29.7, 29.5, 29.4, 26.5, 22.8, 22.8, 14.2, 14.2.

**4-(2-Octyldodecyl)-2-(trimethylstannyl)thiazole (7):** In a 100 mL RBF was added 2-bromo-4-(2-octyldodecyl)thiazole (6) and 20 mL of dry Et2O. The solution was cooled to -78 °C then *n-*BuLi (0.9 mL, 2.5 M) was drop wise added. The reaction was stirred 10 min then TMSnCl (2.92 mL, 1M) was added. The reaction was allowed to reach RT and was quenched with water, extracted with PE and washed with water and brine, dried over Na2SO4 and evaporated giving 1.0 g of yellowish oil. Yield = 85 %.

^1^H NMR (CDCl3, 400 MHz) δ: 6.99 (s, 1H), 2.83 (d, 2H, *J* = 6.90 Hz), 1.84 (m, 1H), 1.25 (m, 32H), 0.88 (t, 6H), 0.44 (s, 9H). ^13^C NMR (CDCl3, 100 MHz) *δ*: 172.7, 160.0, 116.1, 38.1, 36.0, 33.6, 32., 32.1, 32.0, 30.2, 30.2, 29.8, 29.8, 29.8, 29.7, 29.5, 29.5, 26.6, 22.8, 22.8, 14.21, 14.21, -8.0 (3C).

**5,6-Difluoro-4,7-bis(4-(2-octyldodecyl)thiazol-2-yl)benzo[c][1,2,5]thiadiazole (8):** In a 100 mL Schlenck flask was added 4-(2-octyldodecyl)-2-(trimethylstannyl)thiazole (7) (0.8 g, 1.52 mmol), 4,7-dibromo-5,6-difluorobenzo[c][1,2,5]thiadiazole (0.2 g, 0.6 mmol), P(*o*-tolyl)3 (15 mg, 0.049 mmol) and 30 mL of toluene. The solution was degased with a stream of argon and Pd2(dba)3 (11 mg, 0.012 mmol) was added. The flask was capped and stirred for 24h at 120 °C. Then the solvent was removed under vacuum and the residue was purified by chromatography (PE to PE:EtOAc 5%). The waxy residue was then recrystallized twice in hot acetone giving 910 mg of yellow waxy solid. Yield = 67%.

^1^H NMR (CDCl3, 400 MHz) δ: 7.23 (s, 2H), 2.89 (d, 4H, J = 6.83 Hz), 1.94 (m, 2H), 1.28 (m, 64H), 0.8 (t, 12H). ^13^C NMR (CDCl3, 100 MHz) δ: 158.5, 155.0, 151.4 (dd, 2C, J1 = 270.7 Hz, J2 = 19.8 Hz), 148.4, 117.8, 113.0, 112.9, 38.0, 36.2, 33.6, 32.0, 32.0, 30.2, 30.2, 29.8, 29.8, 29. 8, 29. 8, 29. 8, 29.8, 29.5, 29.5, 26.7, 22.8, 22.8, 14.2, 14.2

**4,7-Bis(5-bromo-4-(2-octyldodecyl)thiazol-2-yl)-5,6-difluorobenzo[*c*][1,2,5] thiadiazole (9):** In a 100 mL RBF was added 5,6-difluoro-4,7-bis(4-(2-octyldodecyl)thiazol-2-yl)benzo[c][1,2,5]thiadiazole (8) (260 mg, 0.28 mmol), 30 mL of chloroform and 30 mL of acetic acid. Then NBS (113 mg, 0.64 mmol) was added and the solution was stirred overnight at 30 °C. Then the solution was diluted in 100 mL of water and extracted with chloroform, washed with water and neutralized with NaHCO3 saturated solution. The solvent was removed and the residue was purified by chromatography (CH:Toluene 5 % to CH:Toluene 5%: DCM 10 %) giving 210 mg of yellow waxy solid. Yield = 70 %.

^1^H NMR (CDCl3, 400 MHz) δ: 2.83 (d, 4H, *J* = 7.0 Hz), 2.01 (m, 2H), 1.4-1.18 (m, 64H), 0.86 (td, 12H). ^13^C NMR (CDCl3, 100 MHz) δ: 157.1, 155.0, 151.5 (dd, 2C, *J1* = 273.1 Hz, *J2* = 19.7 Hz), 148.1, 112.6, 112.5, 110.6, 110.6, 38.1, 34.2, 33.6, 33.6, 32.1, 32.1, 30.2, 30.2, 29.9, 29.8, 29.8, 29.8, 29.5, 29.5 26.7, 26.7, 22.9, 22.9, 14.3, 14.3. **HRMS** (ESI-TOF): calcd for C60H98Br2F2N4S3 [M]^+^, 1169.46; found 1169.5328.

**Synthesis of AQM-based monomer**

**Scheme S2.** Synthetic route to AQM-based monomer

**2-dodecyl 1-bromohexadecane (14):** In a round bottom flask, 2-decyl hexadecane 1-ol (10 gr, 24.3 mmol, 1eq) and triphenylphosphine (12.77 gr, 48.6 mmol, 2 eq.) were dissolved in dichloromethane.1 The mixture was cooled to 0 ℃. At this temperature, NBS (6.5 gr, 36.5 mmol, 1.5 eq) was added slowly by portion to the reaction mixture over the period of 30 min. The reaction mixture was let warm to room temperature and stirred for 12 h. The reaction was quenched with water and extracted with dichloromethane. The organic phase was dried over MgSO4, filtered and the crude product was purified by column chromatography to give a colourless liquid (11.2 gr, 90%).

^1^H NMR (300 MHz, CDCl3, ppm): δ = 3.44-3.45 (d, *J* = 4.7 Hz, 2H), 1.56-1.62 (m, 1H), 1.40-1.21 (m, 48H), 0.84-0.91 (m, 6H); ^13^C NMR: δ = 39.7, 39.5, 32.6, 31.9, 29.8, 29.7, 29.7, 29.6, 29.6, 29.4, 26.6, 22.7, 14.1.

**3,6-bis((2-bromothiazol-5-yl) methylene) piperazine-2,5-dione (11):** Into a mixture of 2,5-Piperazinedione (2.3 g, 10 mmol, 1 eq.) and 2-bromothiazole-5-carbaldehyde (5.12 g, 26.68 mmol, 2.3 eq.) in DMF (30 mL), triethylamine was added dropwise via syringe (4.7 g, 46.4 mmol, 4 eq.) at 120 ℃ under N2. Upon addition, the original colourless solution was turned into dark brown, and a brown precipitate was formed during the overnight reaction.2 After cooling to room temperature, the precipitate was collected by filtration and rinsed with acetone. The product was used for the next step without further purification. Yield: 55%.

** (2Z,5Z)-2,5-bis((5-bromothiazole-2-yl) methylene)-3,6-bis(2-dodecylhexadecyloxy)-2,5-dihydropyrazine (12):** A mixture of the 3,6-bis((5-bromothiazole-2-yl) methylene) piperazine-2,5-dione (2) (1.5 g, 3.24 mmol, 1 eq.), K2CO3 (2.23 g, 16.2 mmol, 5 eq.) and alkyl bromide (6.12 g, 13 mmol, 4 eq.) in DMF (15 mL) was stirred at 100 ℃ for 2 hours under N2. After cooling to room temperature, the reaction mixture was filtered, and the filtrate was distilled under reduced pressure. The solid was purified by column chromatography to give an orange solid (42%).

^1^H NMR (300 MHz, CDCl3) δ = 7.74 (s, 1H), 7.08 (s, 1H), 4.35 (d, J = 5.6 Hz, 2H), 1.94 (s, 1H), 1.49 – 1.16 (m, 53H), 0.89 (s, 6H). ^13^C NMR: δ = 158.0, 143.8, 141.8, 137.1, 130.1, 114.0, 37.2, 31.9, 31.6, 30.1, 29.7, 29.66, 29.4, 26.8, 22.7, 14.1. **HRMS** (ESI-TOF): calcd for C68H118Br2N4O2S2 [M]^+^, 1247.65; found 1247.7133.

**Synthesis of thiazolo[5,4-*d*]thiazole monomer**

**Scheme S3.** Synthetic route to unsubstituted TzTz

**2,5-Di(furan-2-yl)thiazolo[5,4-*d*]thiazole (13):** A solution of furan-2-carbaldehyde (3.2 g, 0.033 mol) and dithiooxamide (2.0 g, 0.017 mol) in 50 ml of anhydrous DMF was heated to reflux at 130 °C for 24 h under inert atmosphere. After completion of the reaction, the solvent was evapored and diethyl ether was added (50 ml) to obtain the precipitates. The obtained precipitates were washed by ether and ethanol times. The residue was purified by column chromatography on silica gel with petroleum ether : DCM (1 : 1, v/v) as eluents to give the desired product as greenish solid (2.28 g, 50% yield).

^1^H NMR (CDCl3, 400 MHz) δ: 6.59 (dd, 2H, *J* = 3.6, 2.1 Hz), 7.09 (d, 2H, *J* = 3.3 Hz), 7.56 (d, 2H, *J* = 1.5 Hz).

**Thiazolo[5,4-*d*]thiazole-2,5-dicarboxylic acid (14):** Following a modified literature procedure,^[6]^ 2,5-bis(2-furyl)thiazolothiazole (1.0 g, 3.65 mmol) and 28 ml of pyridine was heated on a water bath with rapid stirring until the solid has dissolved almost completely. After cooling to 70 °C, 10 ml of water was added to produce a uniform suspension of fine crystals. Subsequently, finely powdered of KMnO_4_ (6.34 g, 40 mmol) and 10 ml of H_2_O were added at room temperature. Following this, the mixture was then allowed to react at 40 °C overnight. Then, to the solution, cooled to 25 °C, was introduced 0.85 g of NaHSO_3_ and 10 ml of water. The black precipitate, consisting of manganese dioxide and the potassium salt of the acid, was collected with suction on a large filter and washed with water (150 mL). The filter cake was boiled with 100 mL of water and the extract filtered hot with suction. Two further extractions of the residual manganese dioxide were made with smaller volumes of hot water. The almost colorless filtrates were acidified with concentrated hydrochloric acid and the precipitated acid was collected. The product was washed thoroughly with water, with 1,4-dioxane, and finally with ether. The resultant solid was dried overnight under vacuum to deliver the title compound as a white solid (0.55 g, 70%)

^13^C NMR (CDCl_3_, 100 MHz) δ: 163.8, 161.0, 153.9.

**Thiazolo[5,4-*d*]thiazole (15):** 0.4 g of Thiazolo[5,4-d]thiazole-2,5-dicarboxylic acid (14) was refluxed in EtOH (29 ml) for 48h. Following this, The solvent was removed under reduced pressure and the trituration of the residue with diethylether gave a white crystalline solid (0.24 g, 99%).

^1^H NMR (CDCl3, 400 MHz) δ: 8.93 (s, 2H). ^13^C NMR (CDCl_3_, 100 MHz) δ: 155.3, 150.8. **HRMS** (ESI-TOF): calcd for C4H2N2S2 [M]^+^, 142.19; found 142.9723.

**General procedure for direct (hetero)arylation polymerization.**

To a 10 mL screw cap schlenk flask containing a magnetic stirring bar, Thiazolo[5,4-d]thiazole (TzTz) (100.0 mg, 0.70 mmol, 1.0 equiv), dibrominated heteroaromatic monomer (0.7 mmol, 1.0 equiv), Pd(OAc)_2_ (15.8 mg, 0.07 mmol, 10 mol%), Cu(OAc)_2_ (38.4 mg, 0.21 mmol, 30 mol%), K_2_CO_3_ (243.4 mg, 1.76 mmol, 2.5 equiv) and PPh_3_ (184.7 mg, 0.70 mmol, 1 equiv) were added. The flask was subjected to three cycles of nitrogen-vacuum-nitrogen before adding 3.52 mL (0.2 M) Mesitylene to dissolve the monomers. Then the mixture was degassed and back-filled with nitrogen for five cycles. Subsequently, the vial was placed in a preheated oil bath at 150 °C and the mixture was stirred for 24 h. After cooling to room temperature, the reaction mixture was precipitated into methanol, followed by Soxhlet extraction with methanol, acetone, cyclohexane and chlorobenzene, respectively. The cyclohexane and chlorobenzene fractions were treated with 100 mL of saturated diethyldithiocarbamate solution at 60 °C for 1 h. The organic layer was then washed several times with deionized water and the solvent were removed under vacuum. The polymer was taken off the flask using a spatula and EtOH, it was then filtered on a Teflon membrane and dried under vacuum.

**NMR traces**

**Figure S1.** ^1^H NMR spectrum (CDCl_3_, 400 MHz, 25^o^C) of the 4-Octyl-1-thiocyanatotetradecan-2-one (compound 5).

**Figure S2.** ^13^C NMR spectrum (CDCl_3_, 100 MHz, 25^o^C) of the 4-Octyl-1-thiocyanatotetradecan-2-one (compound 5).

**Figure S3.** ^1^H NMR spectrum (CDCl_3_, 400 MHz, 25^o^C) of the 2-Bromo-4-(2-octyldodecyl)thiazole (compound 6).

**Figure S4.** ^13^C NMR spectrum (CDCl_3_, 100 MHz, 25^o^C) of the 2-Bromo-4-(2-octyldodecyl)thiazole (compound 6).

**Figure S5.** ^1^H NMR spectrum (CDCl_3_, 400 MHz, 25^o^C) of the 4-(2-Octyldodecyl)-2-(trimethylstannyl)thiazole (compound 7).

**Figure S6.** ^13^C NMR spectrum (CDCl_3_, 100 MHz, 25^o^C) of the 4-(2-Octyldodecyl)-2-(trimethylstannyl)thiazole (compound 7).

**Figure S7.** ^1^H NMR spectrum (CDCl_3_, 400 MHz, 25^o^C) of the 5,6-Difluoro-4,7-bis(4-(2-octyldodecyl)thiazol-2-yl)benzo[c][1,2,5]thiadiazole (compound 8).

**Figure S8.** ^13^C NMR spectrum (CDCl_3_, 100 MHz, 25^o^C) of the 5,6-Difluoro-4,7-bis(4-(2-octyldodecyl)thiazol-2-yl)benzo[c][1,2,5]thiadiazole (compound 8).

**Figure S9.** ^1^H NMR spectrum (CDCl_3_, 400 MHz, 25^o^C) of the 4,7-Bis(5-bromo-4-(2-octyldodecyl)thiazol-2-yl)-5,6-difluorobenzo[*c*][1,2,5] thiadiazole (compound 9).

**Figure S10.** ^13^C NMR spectrum (CDCl_3_, 100 MHz, 25^o^C) of the 4,7-Bis(5-bromo-4-(2-octyldodecyl)thiazol-2-yl)-5,6-difluorobenzo[*c*][1,2,5] thiadiazole (compound 9).

**Figure S11.** ^1^H NMR spectrum (CDCl_3_, 400 MHz, 25^o^C) of the (2*Z*,5*Z*)-2,5-bis((5-bromothiazole-2-yl) methylene)-3,6-bis(2-dodecylhexadecyloxy)-2,5-dihydropyrazine (compound 12).

**Figure S12.** ^13^C NMR spectrum (CDCl_3_, 100 MHz, 25^o^C) of the (2*Z*,5*Z*)-2,5-bis((5-bromothiazole-2-yl) methylene)-3,6-bis(2-dodecylhexadecyloxy)-2,5-dihydropyrazine (compound 11).

**Figure S13.** ^1^H NMR spectrum (CDCl_3_, 400 MHz, 25^o^C) of 2,5-di(furan-2-yl)thiazolo[5,4-*d*]thiazole (compound 13).

**Figure S14.** ^13^C NMR spectrum (CDCl_3_, 100 MHz, 25^o^C) of thiazolo[5,4-*d*]thiazole-2,5-dicarboxylic acid (compound 14).

**Figure S15.** ^1^H NMR spectrum (CDCl_3_, 400 MHz, 25^o^C) of thiazolo[5,4-*d*]thiazole (compound 15).

**Figure S16.** ^13^C NMR spectrum (CDCl_3_, 100 MHz, 25^o^C) of thiazolo[5,4-*d*]thiazole (compound 15).

1. **SEC data**

**Table S1.** Molar masses and dispersity.

| **Polymer** | **F°. Cyclohexane** | | **F°. CB** | | | **F°. TCB** | | Total Yield (%) |
| --- | --- | --- | --- | --- | --- | --- | --- | --- |
|  | Yield (%) | M_n_/M_w_ (kDa) | Yield. (%) | M_n_/M_w_ (kDa) | Đ | Yield. (%) | M_n_/M_w_ (kDa) |  |
| **PBT-TzTz** | **--** | **--** | 77 | **14.5/37.6** | 2.6 | **--** | **--** | 77 |
| **PQx-TzTz** | **--** | **--** | 29 | 3.3/7.1 | 2.1 | 29 | 2.9/4.2 | 58 |
| **PTPD-TzTz** | **--** | **--** | 71 | **7.3/13.9** | 1.9 | **--** | **--** | 71 |
| **PBTD-TzTz** | 20 | 2.8/11.1 | 78 | **10.3/16.4** | 1.6 | **--** | **--** | 98 |
| **PIID-TzTz** | 68 | **5.4/13.3** | 31 | 1.5/6.6 | 4.4 | **--** | **--** | 99 |
| **PAQM-TzTz** | 20 | 3.6/5.7 | 22 | **3.9/7.4** | 1.9 | 23 | 3.8/5.3 | 65 |
| **PDPP-TzTz** | **--** | **--** | 53 | **3.9/8.3** | 2.1 | **--** | **--** | 53 |

In bold, the highest measured molar masses for each polymer.


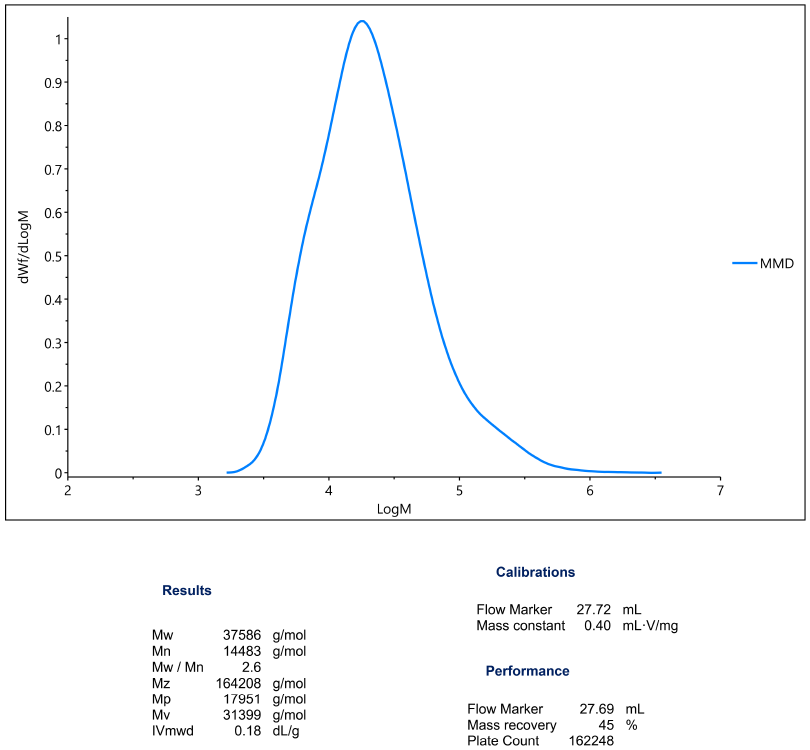


**Figure S17.** SEC Trace of PBT-TzTz (Fr. ClBn).


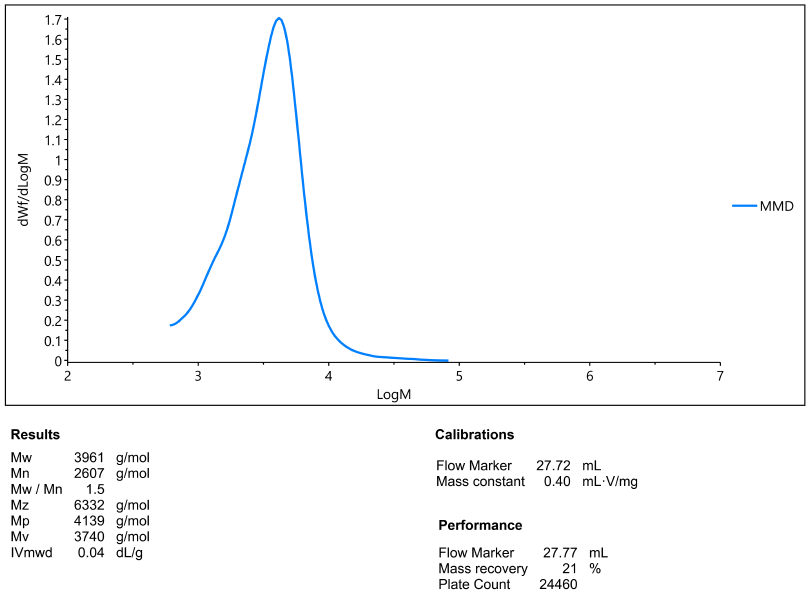


**Figure S18.** SEC Trace of PQx-TzTz (Fr. ClBn).


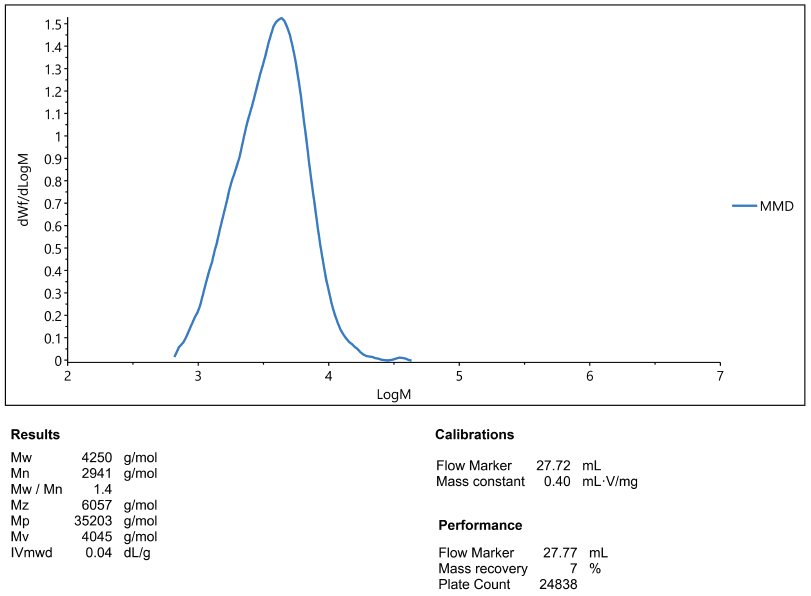


**Figure S19.** SEC Trace of PQx-TzTz (Fr. 3ClBn).


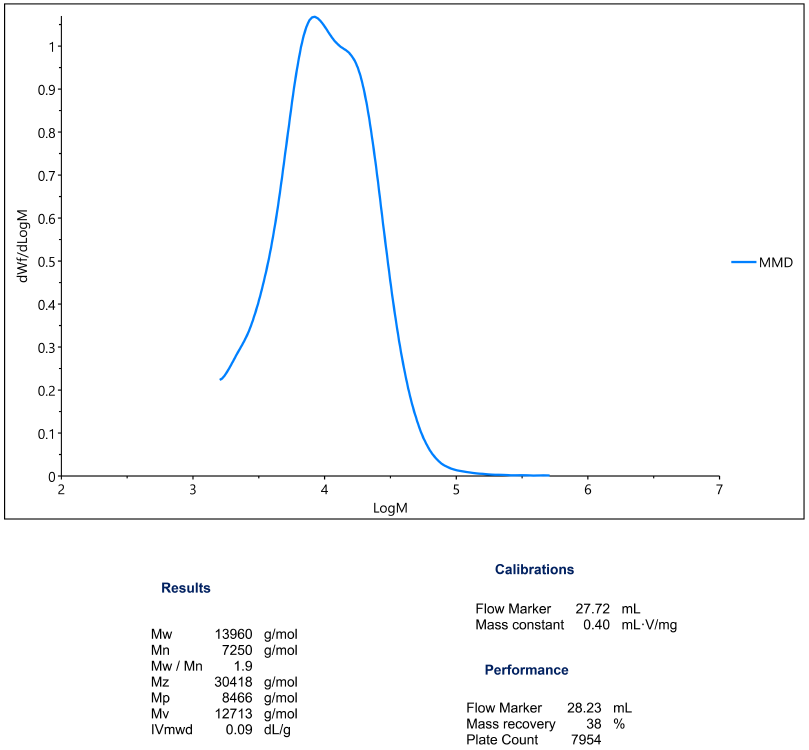


**Figure S20.** SEC Trace of PTPD-TzTz (Fr. ClBn).


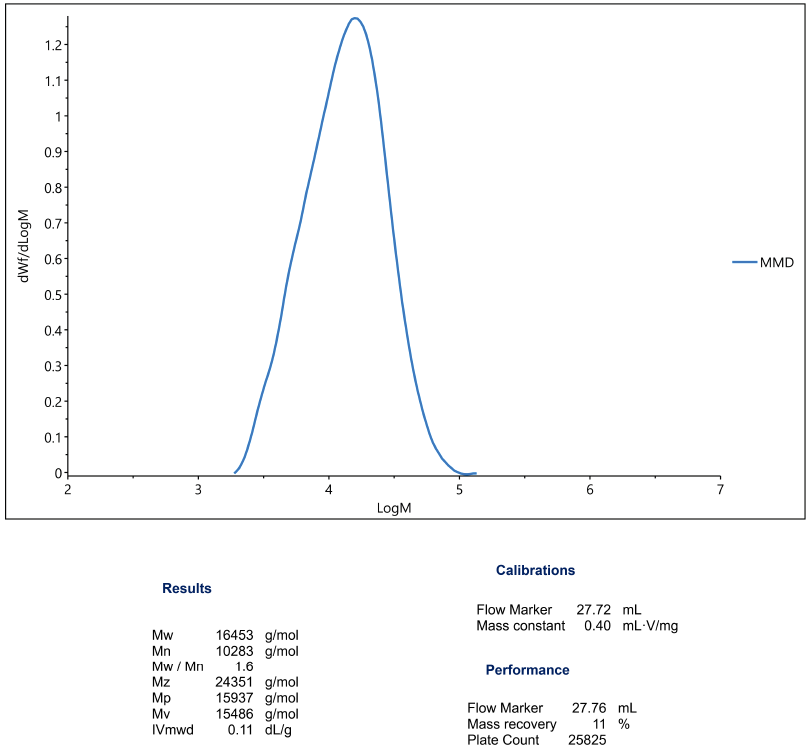


**Figure S21.** SEC Trace of PBTD-TzTz (Fr. ClBn).


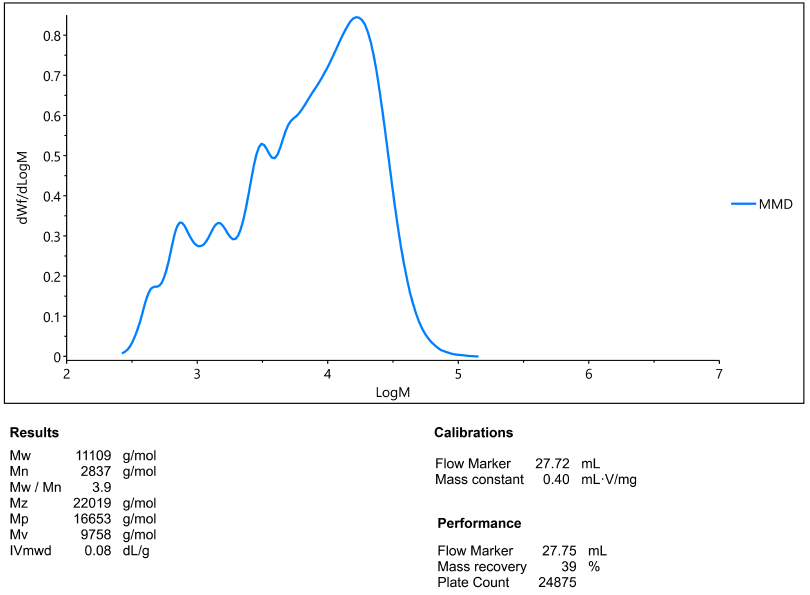


**Figure S22.** SEC Trace of PBTD-TzTz (Fr. Cyclo).


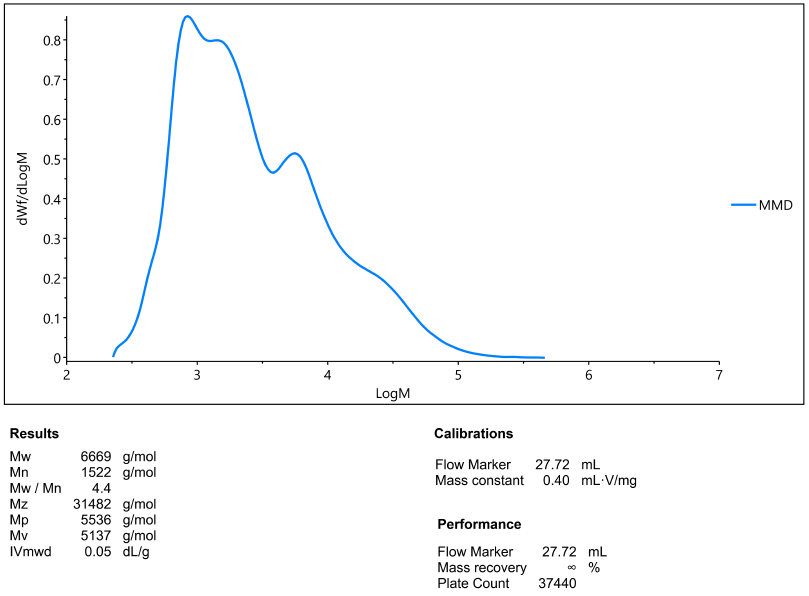


**Figure S23.** SEC Trace of PIID-TzTz (Fr. ClBn).


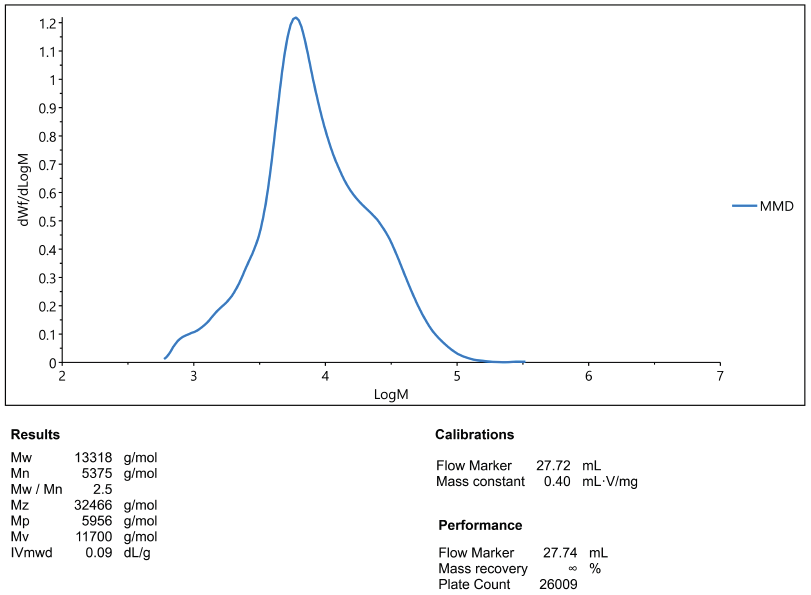


**Figure S24.** SEC Trace of PIID-TzTz (Fr. Cyclo).


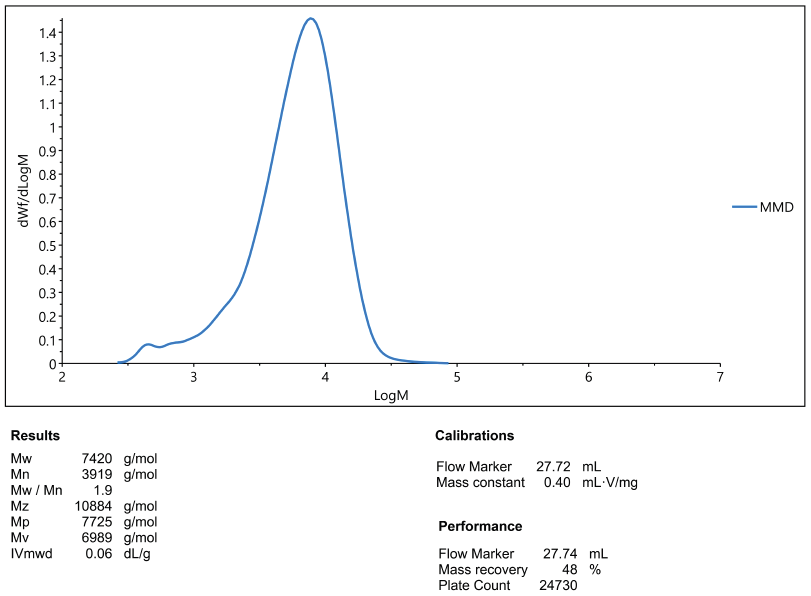


**Figure S25.** SEC Trace of PAQM-TzTz (Fr. ClBn).


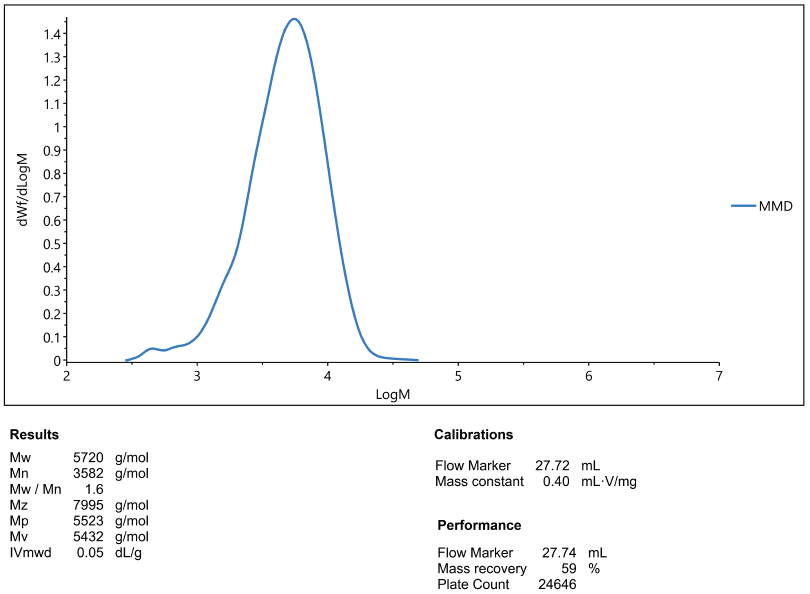


**Figure S26.** SEC Trace of PAQM-TzTz (Fr. Cyclo).


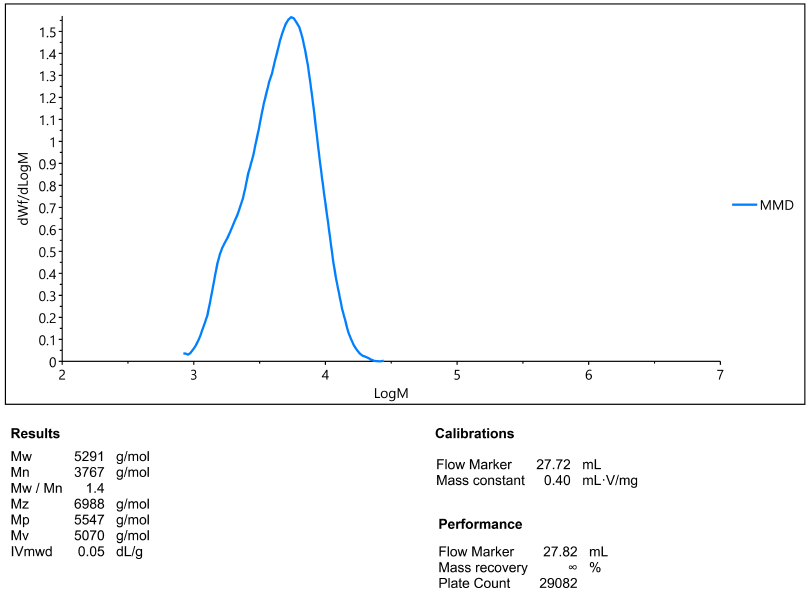


**Figure S27.** SEC Trace of PAQM-TzTz (Fr. 3ClBn).


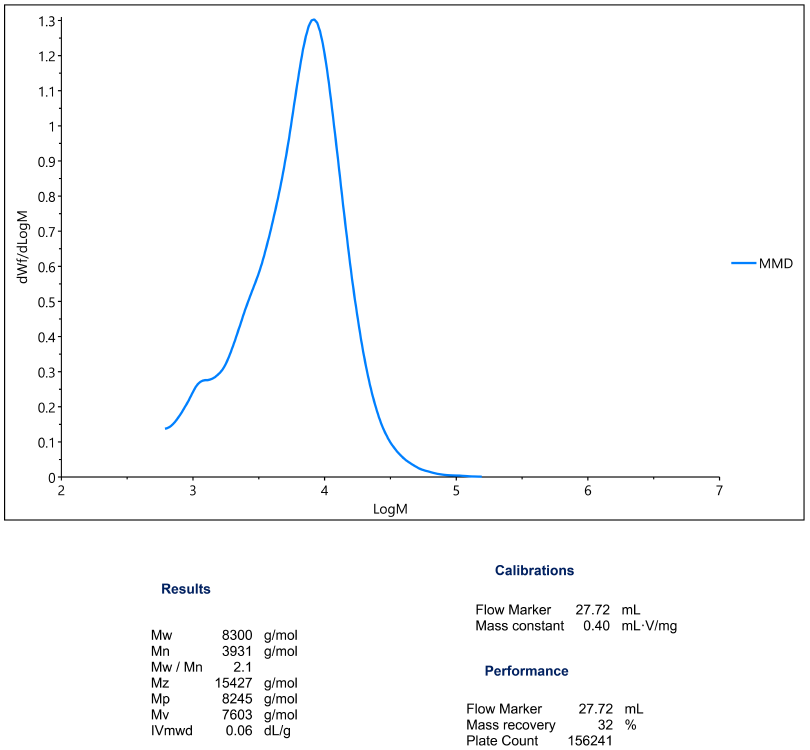


**Figure S28.** SEC Trace of PDPP-TzTz (Fr. ClBn).

1. **Thermal characterizations**

**Table S2:** starting decomposition temperature from TGA (Q50 from TA Instruments; conditions: 5°C/min, N2) and initial solvent content from weight loss between 140 and 270°C.

| **Polymer** | **T_dec_ (°C)^a^** | **% solvent^b^** |
| --- | --- | --- |
| **PBT-TzTz** | 320 | 0.9 |
| **PQx-TzTz** | 360 | 10.0 |
| **PTPD-TzTz** | 330-350 | 10.7 |
| **PDFBTz-TzTz** | 330 | 4.0 |
| **PIID-TzTz** | 340 | 28.2 |
| **PAQM-TzTz** | 300 | 6.7 |
| **^a^ T_dec_:** starting decomposition temperature from TGA; **^b^** **% solvent:** weight content of solvent from TGA. | | |

**Figure S29:** DSC heating-cooling cycle after preheating the polymers at 250°C under N2 flow for drying; DSC Q1000 from TA Instruments, 5°C/min, endotherm up.

**PBT-TzTz**


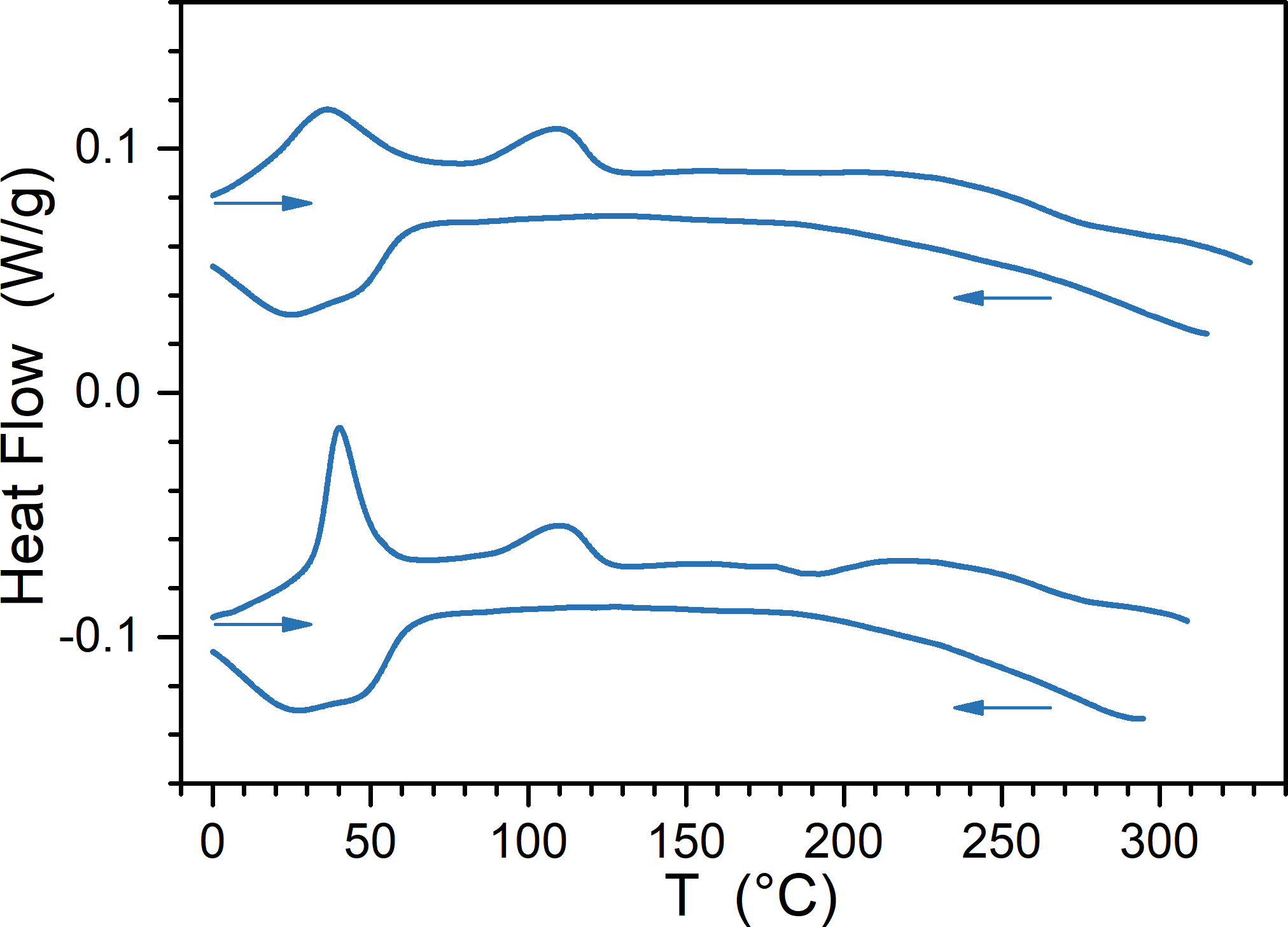


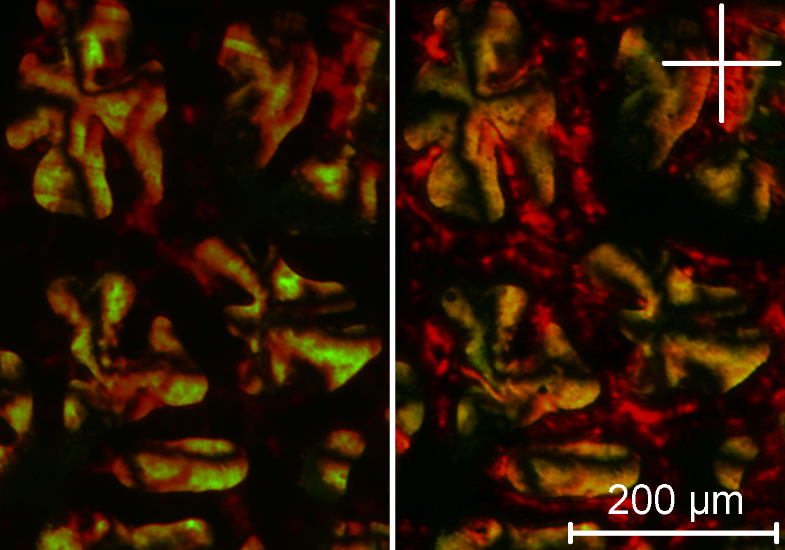


**Figure S30.** **Top:** DSC curves of the pristine PBT-TzTz polymer on first heating and cooling (bottom) and heating-cooling DSC cycle after preheating the polymer at 250°C under N2 flow for trace solvent removal (top). **Bottom:** POM textures of the polymer at 180°C and 300°C, the directions of the crossed polarizers being given by the white cross.

The PBT-TzTz polymer is semi-crystalline at room temperature (r.t.), shows a solid phase transformation just above r.t. and melts to a fluid and birefringent liquid crystalline phase at 90-110°C. The liquid crystalline phase is maintained at least up to 320°C, which is the starting sample degradation temperature determined by TGA at 5°C/min under N2 flow.


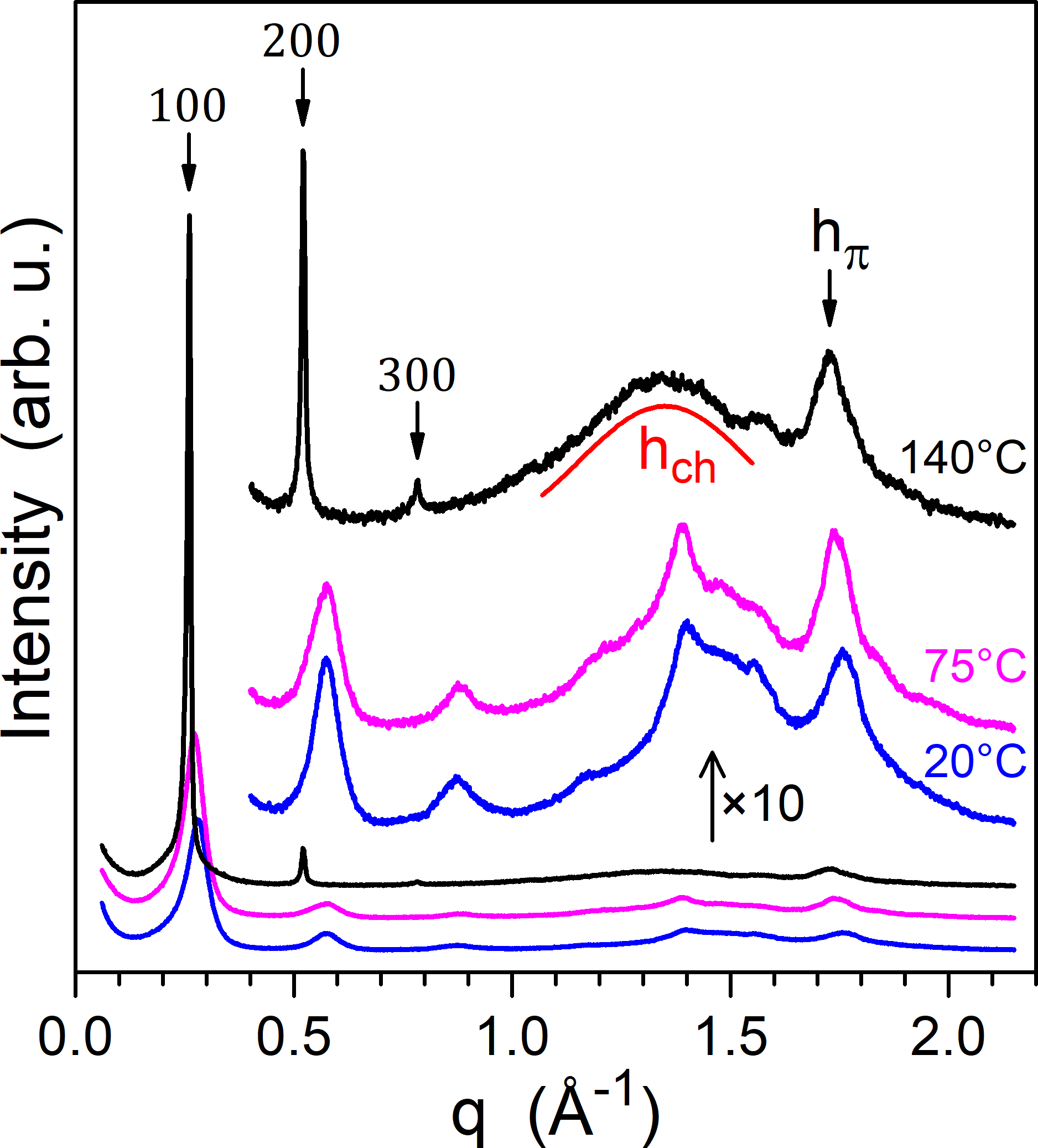


**Figure S31.** SWAXS (Small and Wide-Angle X-ray Scattering) patterns of PBT-TzTz polymer at 20°C, 75°C and 140°C.

The patterns confirm that the two low-temperature phases of PBT-TzTz polymer are semi-crystalline solid states characterized by: i) a complex scattering profile in the 1.3-1.55 Å^-1^ *q*-range that reveals the crystallized state of the linear alkyl side-chains, ii) a scattering maximum h_p_ at *q* ≈ 1.75 Å^-1^ from the p-stacking of backbones into layers (20°C: *h*_p_ = 3.57 Å, correlation length x = 6 nm, as calculated from Scherrer equation with K = 0.9; 75°C: *h*_p_ = 3.61 Å, x = 7 nm) and iii) a series of *h*00 reflections of the lamellar periodicity generated by the alternation of backbone and side-chain layers (20°C: *d*_lam_ = 22.4 Å, x = 12 nm; 75°C: *d*_lam_ = 23.1 Å, x = 12 nm). The high-temperature phase is a fluid smectic-like mesophase, as shown by the broad scattering maximum h_ch_ at *q* ≈ 1.4 Å^-1^ from lateral distances between molten side-chains (*h*_ch_ = 4.6 Å, x = 1 nm), the unchanged signature of backbone stacking (*h*_p_ = 3.63 Å, x = 7 nm) and the lamellar periodicity correlated to longer range due to the release of steric constrains affecting the molecular self-assembly in the crystallized states (*d*_lam_ = 24.1 Å, x ≥ 100 nm).

1. **UV-vis Absorbance**

**Figure S32.** Absorbance UV-vis spectra of **PBT-TzTz** in solution (full line) and in thin-film (dashed line).

**Figure S33.** Absorbance UV-vis spectra of **PQx-TzTz** in solution (full line) and in thin-film (dashed line).

**Figure S34.** Absorbance UV-vis spectra of **PTPD-TzTz** in solution (full line) and in thin-film (dashed line).

**Figure S35.** Absorbance UV-vis spectra of **PBTD-TzTz** in solution (full line) and in thin-film (dashed line).

**Figure S36.** Absorbance UV-vis spectra of **PIID-TzTz** in solution (full line) and in thin-film (dashed line).

**Figure S37.** Absorbance UV-vis spectra of **PAQM-TzTz** in solution (full line) and in thin-film (dashed line).

**Figure S38.** Absorbance UV-vis spectra of **PDPP-TzTz** in solution (full line) and in thin-film (dashed line).

**Figure S39.** Absorbance UV-vis spectra of **PBTTT-C_14_** in solution (full line) and in thin-film (dashed line).

1. **Cyclic Voltametry**

**PBT-TzTz**

**Figure S40.** Reduction scan obtained for P**BT**-TzTz thin-film by cyclic voltammetry in Acetonitrile.

**PQx-TzTz**

**Figure S41.** Reduction scan obtained for P**Qx**-TzTz thin-film by cyclic voltammetry in Acetonitrile.

**PTPD-TzTz**

**Figure S42.** Reduction scan obtained for P**TPD**-TzTz thin-film by cyclic voltammetry in Acetonitrile.

**PBTD-TzTz**

**Figure S43.** Reduction scan obtained for P**BTD**-TzTz thin-film by cyclic voltammetry in Acetonitrile.

**PIID-TzTz**

**Figure S44.** Reduction scan obtained for P**IID**-TzTz thin-film by cyclic voltammetry in Acetonitrile.

**PAQM-TzTz**

**Figure S45.** Reduction scan obtained for P**AQM**-TzTz thin-film by cyclic voltammetry in Acetonitrile.

**PDPP-TzTz**

**Figure S46.** Reduction scan obtained for P**DPP**-TzTz thin-film by cyclic voltammetry in Acetonitrile.

1. **PESA**

**Figure S47.** Photoelectron spectroscopy in air (PESA) spectra of all seven copolymers.

1. **DFT calculations**

**Figure S48.** Schematic representation of the angle measured by DFT between the two ends of the molecular models, after energy minimisation.


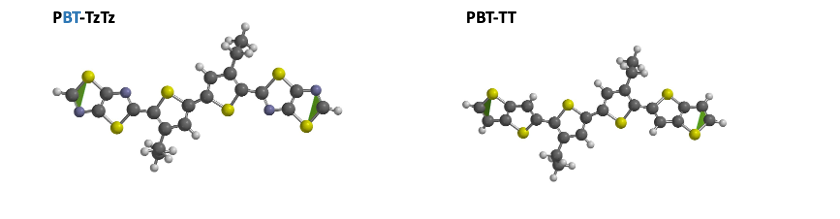


**Figure S49.** Planarity of PBT-TzTz (left: 9°) and PBT-TT (right: 65°).


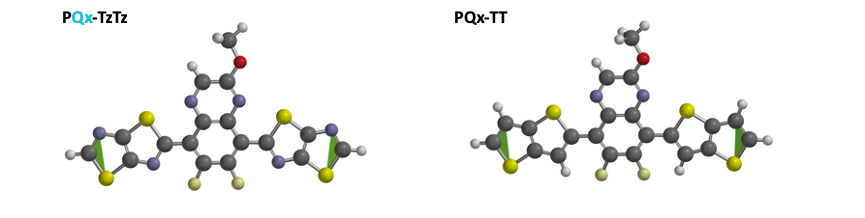


**Figure S50.** Planarity of PQx-TzTz (left: 0°) and PQx-TT (right: 0°).


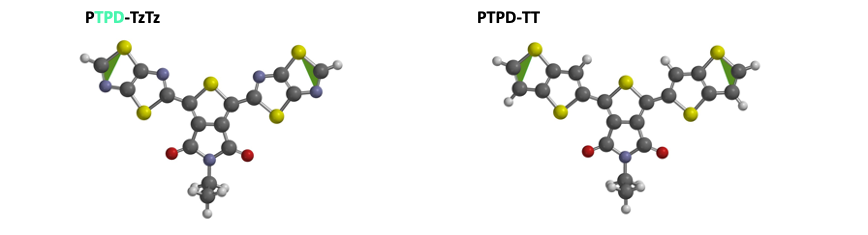


**Figure S51.** Planarity of PTPD-TzTz (left: 0°) and PTPD-TT (right: 1°).


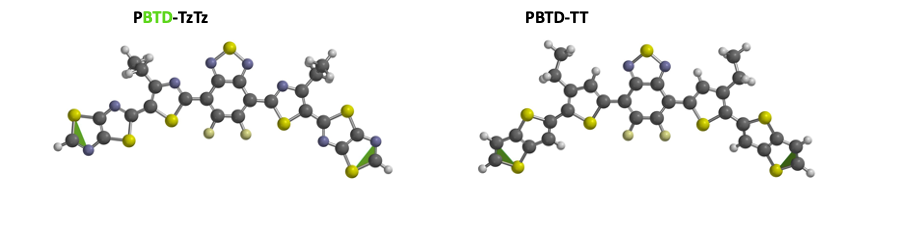


**Figure S52.** Planarity of PBTD-TzTz (left: 15°) and PBTD-TT (right: 29°).


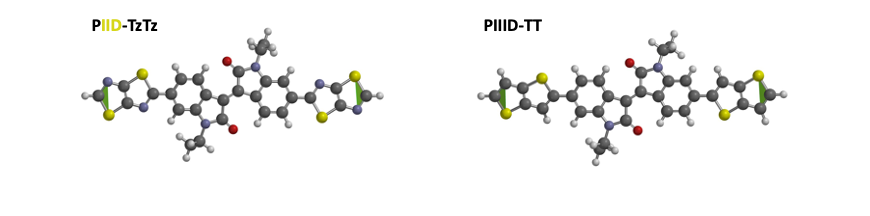


**Figure S53.** Planarity of PIID-TzTz (left: 14°) and PIID-TT (right: 18°).


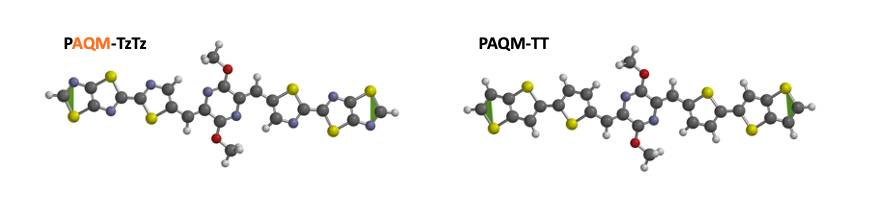


**Figure S54.** Planarity of PAQM-TzTz (left: 0°) and PAQM-TT (right: 0°).


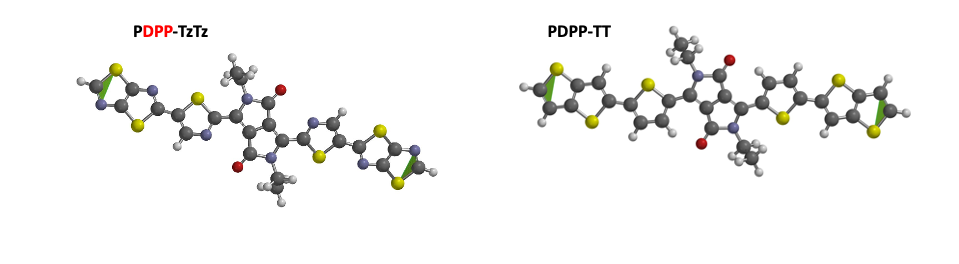


**Figure S55.** Planarity of PDPP-TzTz (left: 40°) and PDPP-TT (right: 9°).

1. **References**

[1] V. Vijayakumar, E. Zaborova, L. Biniek, H. Zeng, L. Herrmann, A. Carvalho, O. Boyron, N. Leclerc, M. Brinkmann, *ACS Appl. Mater. Interfaces* **2019**, *11*, 4942-4953.

[2] T. Olla, R. Jabbour, A. Labiod, O. Boyron, S. Méry, B. Heinrich, T. Heiser, D. Jacquemin, P. Lévêque, A. Lesage, N. Leclerc, *Adv. Funct. Mater*. **2022**, *32*, 2204929.

[3] P. Chávez, I. Bulut, S. Fall, O. A. Ibraikulov, C. L. Chochos, J. Bartringer, T. Heiser, P. Lévêque, N. Leclerc, *Molecules* **2018**, *23*, 1270.

[4] L. Ma, B. Chen, Y. Guo, Y. Liang, D. Zeng, X. Zhan, Y. Liu, X. Chen, *J. Mater. Chem. C* **2018**, 6, 13049-13058.

[5] M. Kuramochi, J. Kuwabara, W. Lu, T. Kanbara, *Macromolecules*, **2014** 47, 7378-7385.

[6] J. R. Johnson, D. H. Rotenberg, R. Ketcham, *J. Am. Chem. Soc.* **1970**, 92, 4046.
